# Intravenous Foreign Body at the Hand: Case Report

**DOI:** 10.1055/s-0042-1743522

**Published:** 2022-03-03

**Authors:** Jose Couceiro, Elena Garcia-Valladares, Jose Fernandez-Divar, Manuel Sanchez-Crespo, Higinio Ayala, Fernanado Del Canto

**Affiliations:** 1 Department of Orthopedics, Hospital Universitario Marques de Valdecilla, Santander, Spain

**Keywords:** intravenous, foreign body, hand

## Abstract

Intravenous foreign bodies following trauma to the hand are relatively uncommon with sparse reports of this condition being published in the literature. They have been reported to migrate as far as the thoracic cavity and the heart. In the following case report, we describe a case of an intravenous foreign body following hand trauma, and the treatment and potential complications are also discussed.

Intravenous foreign bodies following a hand trauma are relatively uncommon with sparse reports of this condition being published in the literature. 1 2 3 4 They have been reported to migrate as far as the thoracic cavity and the heart. 1 In the present case report, we describe a case of an intravenous foreign body following hand trauma, and the treatment and potential complications are also discussed.

## Methods

**Fig.1:**
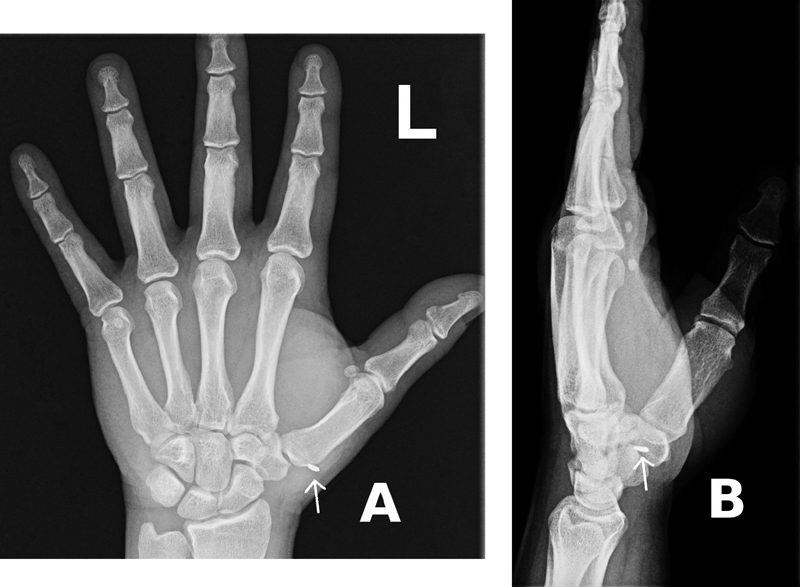
Radiolucent foreign body was detected on simple X-rays. (A) Anteroposterior and (B) profile of the patient's left hand (white arrows).

Our patient is a 37-year-old man, a manual worker who injured his hand while working with two hammers, he was not wearing gloves or other protective equipment, one of the hammers hit the other, and a piece of metal produced a puncture wound on his left hand. The patient's neurological examination was unremarkable, and he had no signs of tendinous involvement. There was no active bleeding or pulsatile mass, a small hematoma was visible, and the patient referred having bled profusely at the accident site. A small metallic foreign body was detected on simple X-rays ( Fig. 1 ). Attempts at extraction in the emergency room were unsuccessful.

**Fig. 2:**
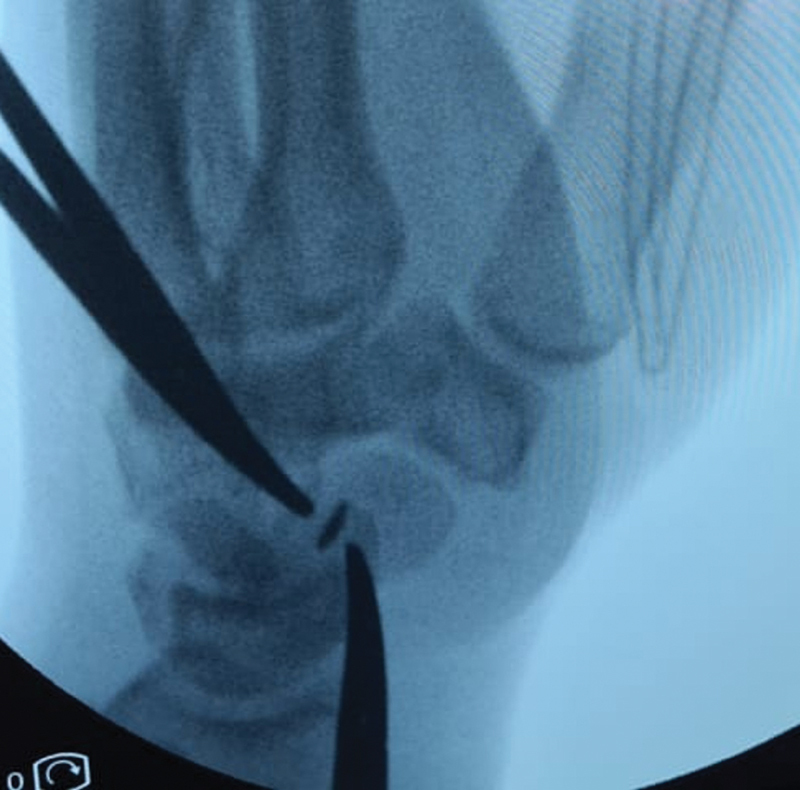
The foreign body was located using two mosquitoes.

**Fig. 3:**
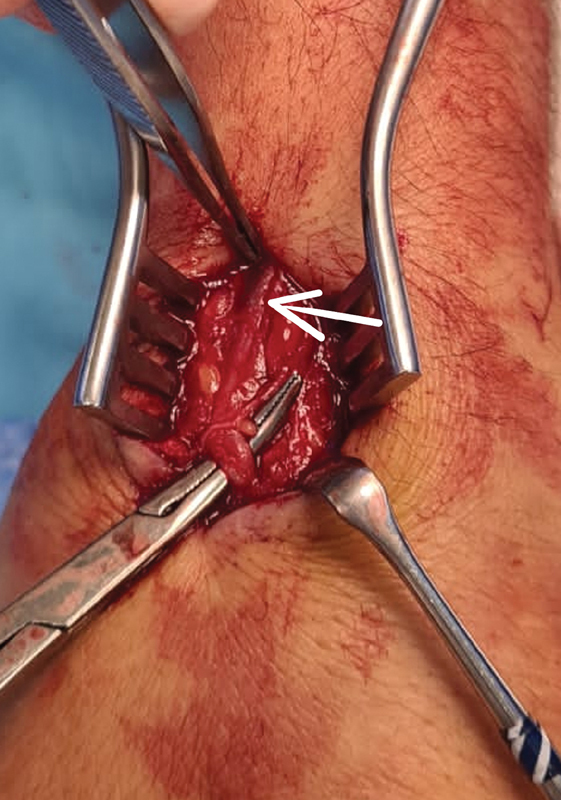
The foreign body appeared as a black spot inside of the superficial vein, close to the pickups in the picture (white arrow); the entry site was just on top of the mosquito.

The patient was taken for surgery, which was conducted under a brachial plexus block; the wound was extended proximally. Intraoperatively, we found that the metal piece had opened one of the radial superficial veins at the puncture wound and was located inside of the lumen ( Figs. 2 and 3 ), surrounded by a small thrombus. A mosquito was placed proximal to the foreign body to prevent migration and the injured vein was excised along with the foreign body.

## Results

The postoperative course was uneventful and the patient was back to work in 3 weeks.

## Discussion

Most intravascular foreign bodies are iatrogenic, and these include cannulas, stents, and needles among others. 5 6 Posttraumatic intravenous foreign bodies are uncommon, and their migration to the thoracic cavity has an estimated 2% mortality rate. 1 Migrating foreign bodies are more common in the military or civilian setting after penetrating firearm injuries or explosions. 2 4 They can occur after work or leisure-related injuries, however. 2 3

The common patient history involves a puncture wound following some work with two metal pieces colliding against each other. A small metal shard shoots out and produces a puncture wound on the patient's hand, causing profuse bleeding; patients are commonly asymptomatic otherwise.

X-rays are the preferred imaging study for the initial diagnosis. When migration is suspected, other imaging techniques such as ultrasound or computed tomography can be used to locate the foreign body, ultrasound can be especially useful if the foreign body is not metallic in nature or to ascertain patency of the foramen ovale should migration to the heart occur. 1

Metallic foreign bodies can be detected with the image intensifier and are usually seen as a “black spot” inside of the vessel. In our case, we triangulated the foreign body's position with two mosquitoes. Foreign body retrieval and vessel repair or ligation and excision have been reported. 1 2 3 4 If the foreign body is retrieved previously to intrathoracic migration, complications are relatively rare. 1 2 In our case, the foreign body was superficial and had not migrated, but should the access become more difficult minimally invasive endovascular techniques with special snares, loops, baskets, and other instruments have been described with favorable outcomes and minimal morbidity to the patient. 6

Should the foreign body migrate to the heart consultation with cardiothoracic surgery is recommended, 1 3 complications in this scenario include embolization, cardiac damage, arrhythmias, valvular malfunction endocarditis, cardiac neurosis, and even death. 2 In addition, 6% of patients have a patent foramen ovale and an intravenous foreign body may migrate to the arterial system. 1 Foreign bodies at the heart level can be removed openly via thoracotomy or minimally invasively by the interventional radiology department.
